# Epidemiological characteristics and risk factors of high-risk HPV infection, cervical cancer, and precancerous lesions among women in Southwestern China

**DOI:** 10.3389/fonc.2025.1645748

**Published:** 2025-09-09

**Authors:** Lu Zhang, Qiwen Zhang, Bing Pang, Zhuo Tan, Denghui Yang, Jieru Peng, Yao Dong, Xia Wu, Liu Yang, Youlin Qiao, Chunxia Yang

**Affiliations:** ^1^ Department of Epidemiology and Biostatistics, West China School of Public Health and West China Fourth Hospital, Sichuan University, Chengdu, Sichuan, China; ^2^ Department of Medical Records Statistics, Chengdu Women’s and Children’s Central Hospital, School of Medicine, University of Electronic Science and Technology of China, Chengdu, Sichuan, China; ^3^ Healthcare Department, Chengdu Women’s and Children’s Central Hospital, School of Medicine, University of Electronic Science and Technology of China, Chengdu, Sichuan, China; ^4^ Chinese Academy of Medical Sciences, and Peking Union Medical College, School of Population Medicine and Public Health, Beijing, China

**Keywords:** cervical cancer, human papillomavirus infection, screening, epidemiology, risk factor

## Abstract

**Background:**

Cervical cancer poses a significant threat to women’s reproductive and overall health. In Chengdu, southwestern China, free cervical cancer screening is provided to women in both urban and suburban areas, using high-risk human papillomavirus (HR-HPV) testing combined with cytology triage. This study aimed to investigate the epidemiological characteristics and risk factors of HR-HPV infection, cervical cancer, and high-grade precancerous lesions based on large-scale screening data from Chengdu.

**Methods:**

This retrospective study analyzed cervical cancer screening data from January 1, 2021, to December 31, 2022, in Chengdu. A total of 107,120 women aged 35–64 years who underwent screening with HR-HPV testing combined with cytology triage were included. Screening participation and detection outcomes were analyzed to evaluate program implementation and to describe the distribution of HR-HPV and cervical lesions. Multivariable logistic regression was performed to identify factors independently associated with HR-HPV infection and cervical cancer/precancerous lesions.

**Results:**

The overall prevalence of HR-HPV infection was 10.54%, with HPV 16/18 accounting for 1.26%. The crude detection rate of cervical cancer and high-grade precancerous lesions (≥ HSIL/CIN2-3) was 399.55 per 100,000, of which the detection rate of cervical cancer was 19.60 per 100,000. The early diagnosis rate through screening reached 97.66%. The distribution of HPV 16/18 and other HR-HPV types varied across different cervical lesion groups, with HPV 16/18 being the predominant types associated with cervical cancer. Multivariable logistic regression analysis showed that age ≥55 years (55–59 years: aOR=1.34, 95% CI: 1.22-1.47; 60–64 years: aOR=1.53, 95% CI: 1.37-1.73), residence in suburban areas (aOR=1.19, 95% CI: 1.11-1.27), menopause (aOR=1.08, 95% CI: 1.01-1.15), having three or more childbirths (aOR=1.18, 95% CI: 1.06-1.31), and three or more abortions (aOR=1.16, 95% CI: 1.06-1.26) were associated with a higher risk of HR-HPV infection. In contrast, later age at first birth (21–25 years: aOR=0.88, 95% CI: 0.83-0.94; ≥26 years: aOR=0.80, 95% CI: 0.74-0.86) and condom use (aOR=0.87, 95% CI: 0.82-0.92) were protective factors. Additionally, age (55–59 years: aOR=1.57, 95% CI: 1.03-2.41), residential areas (suburban areas: aOR=1.82, 95% CI: 1.23-2.69), and menopausal status (yes or uncertainty: aOR=0.72, 95% CI: 0.52-0.99) were also associated with cervical cancer and precancerous lesions. A potential interaction between age and menopausal status was also observed.

**Conclusion:**

This study characterized the epidemiology of HR-HPV infection, cervical cancer, and high-grade precancerous lesions in Chengdu and identified associated risk and protective factors, providing evidence to inform targeted screening and prevention strategies.

## 1 Introduction

Cervical cancer is a malignant tumor that develops in the cervix and represents the most common malignancy of the female reproductive system (1). According to the International Agency for Research on Cancer (IARC), approximately 660,000 new cases and 350,000 deaths from cervical cancer were reported globally in 2022, with China accounting for 22.8% of the global incidence and 16.1% of the mortality. In recent years, the incidence and mortality of cervical cancer in China have shown an upward trend, accompanied by a shift toward younger age at onset. Substantial regional disparities persist, with higher disease burden observed in rural compared with urban areas, and in central and western regions compared with the eastern region (2). Cervical cancer poses a serious threat to women’s reproductive health and remains a pressing public health and societal challenge in China.

Human papillomavirus (HPV) infection is the most common sexually transmitted infection globally (3). Persistent infection with high-risk HPV (HR-HPV) types is recognized as the primary cause of cervical cancer, with nearly 99.7% of cases linked to HR-HPV infection (3, 4). In China, HPV infection rate among women varies markedly across regions, ranging from 6.2% to 31.3% (5). The reported prevalence of HPV infection in Sichuan is 23.84%, higher than the national average of 15.54% (5, 6).

Prophylactic HPV vaccination and cervical cancer screening are recognized strategies for cervical cancer prevention. HPV vaccination is an effective primary prevention strategy, protecting against most HR-HPV infections that cause cervical cancer. The bivalent, quadrivalent, and nine-valent HPV vaccines were approved in China in 2016, 2017, and 2018, but HPV vaccination is not yet included in the National Immunization Program (NIP) and remains self-paid, resulting in relatively low coverage (7). Cervical cancer screening allows early detection of precancerous lesions, enabling timely intervention and reducing both incidence and mortality. Common screening methods include cytology and HPV DNA testing. Cytology identifies abnormal cells based on morphological changes, whereas HPV testing detects viral DNA at the molecular level (8). Recently, the combined screening approach of cytology and HPV testing has gained prominence due to their complementary sensitivity and specificity in accurately detecting cervical precancerous lesions and early-stage cancer (9, 10). However, cytology remains the main primary screening method in most regions of China, and large-scale HPV-based screening is still limited. As a result, most studies have focused on small community trials or limited populations, with few data from large-scale screening programs.

In response to the WHO’s initiative to eliminate cervical cancer and China’s Healthy China Action Plan (2019–2030), Chengdu, the capital city of Sichuan Province, launched the Healthy City Project in 2021, which includes the Pilot Program for Comprehensive Cervical Cancer Prevention and Control. This program provides subsidies for HPV vaccination among girls aged 13–14 years and offers free cervical cancer screening based on HR-HPV testing combined with cytology triage for women aged 35–64 years. Based on the extensive population data from the screening program, this study aims to analyze the epidemiological characteristics and risk factors of HR-HPV infection, cervical cancer, and high-grade precancerous lesions among women in southwestern China, providing scientific evidence for developing regional prevention and control strategies.

## 2 Methods

### 2.1 Data source and participants

This retrospective study analyzed cervical cancer screening results from January 1, 2021, to December 31, 2022, in Chengdu. Data were obtained from the Cervical Cancer Screening Registration Database, with approval from the local health administrative department. The screening program targeted women aged 35–64 years who had a history of sexual activity, were informed about the screening procedures, voluntarily agreed to participate, and provided written informed consent. Exclusion criteria included a history of cervical cancer, hysterectomy, or cervical surgery; pregnancy or menstruation; and use of vaginal medication or sexual activity within 72 hours prior to screening.

**Figure 1:**
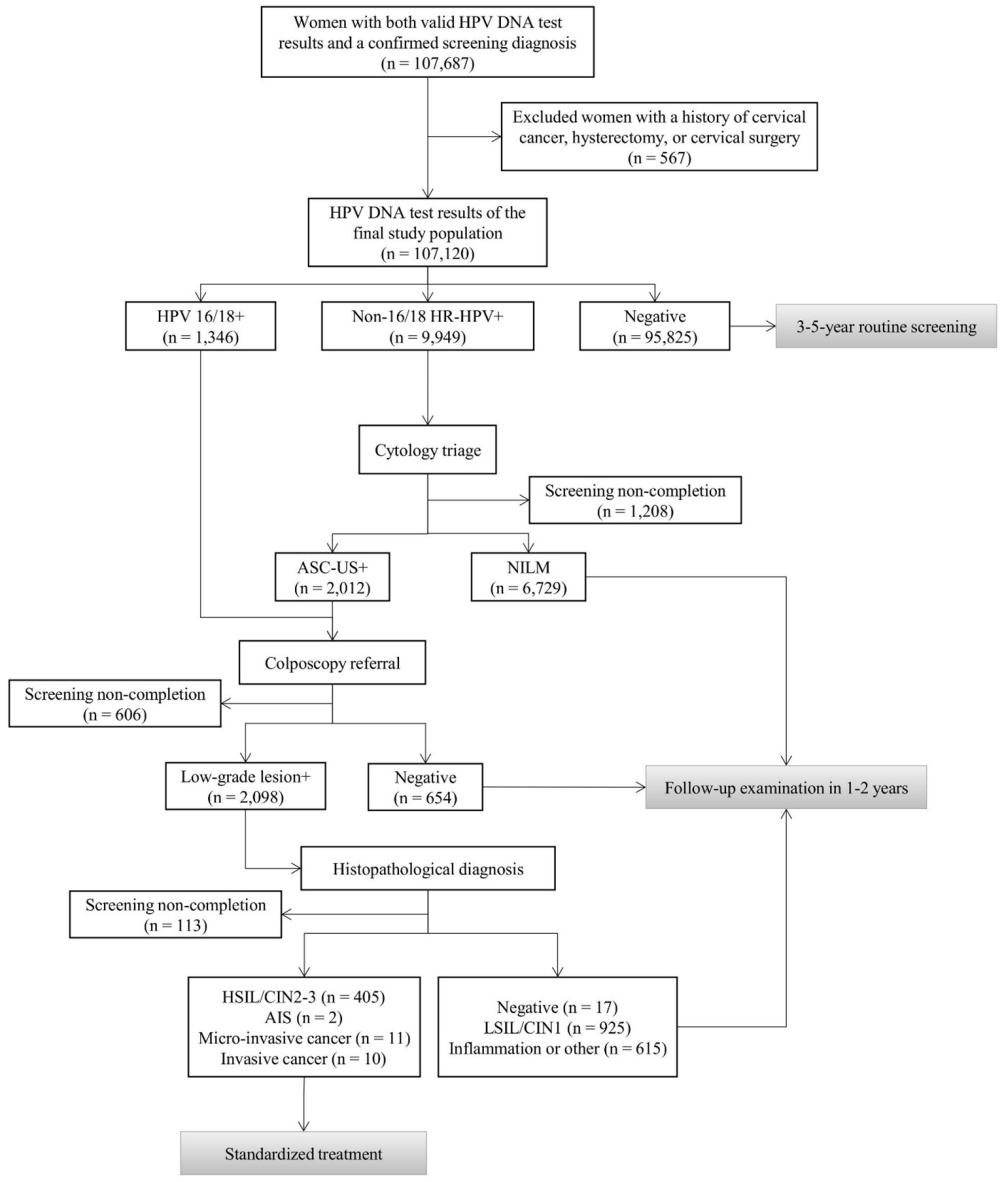
The HPV-based cervical cancer screening workflow.

A total of 107,687 participants with valid HPV DNA test results and a confirmed screening diagnosis in the database were initially enrolled in this study. After excluding 567 women with a history of cervical cancer, hysterectomy, or cervical surgery, 107,120 participants were included in the final analysis (**Figure 1**).

This study was approved by the Ethics Committee of West China Fourth Hospital and West China School of Public Health, Sichuan University (No. Gwll2023141). All participant’s data we accessed and analyzed were completely anonymous and did not contain information that could identify individual participants, thus the requirement for informed consent was waived by the ethics committee.

### 2.2 Screening strategies

Designated hospitals and primary maternal and child health care hospitals in Chengdu were responsible for implementing the free cervical cancer screening program. Women participating in the screening first underwent routine gynecological examinations, followed by cervical specimen collection performed by trained gynecologists. The collected specimens were stored under appropriate conditions and transported to designated laboratories for HPV testing within the specified timeframe.

HPV testing was conducted using PCR-based assays with the High-Risk Human Papillomavirus Nucleic Acid Detection Kit (Fluorescent PCR method, Guangdong Hybribio Biotech Co., Ltd). The kit detects at least 13 HR-HPV genotypes (16, 18, 31, 33, 35, 39, 45, 51, 52, 56, 58, 59, and 68), and allows individual reporting of types 16 and 18. All procedures for specimen collection, storage, and laboratory testing were conducted according to the manufacturers’ instructions.

**Figure 1** illustrates the detailed HPV-based cervical cancer screening workflow. Participants who tested positive for HPV types 16 or 18 were directly referred for colposcopy. Those infected with non-16/18 HR-HPV types underwent cytology triage, and individuals with abnormal cytology results were subsequently referred for colposcopy. Finally, participants with abnormal colposcopic findings were required to undergo histopathological examination. If diagnosed with high-grade squamous intraepithelial lesions (HSIL) or worse, they were promptly informed and referred for appropriate treatment.

### 2.3 Explanation of the screening results

For the HPV-based screening strategy, women who tested positive for HPV 16/18, or those positive for non-16/18 HR-HPV combined with cytology results of atypical squamous cells of undetermined significance (ASC-US) or worse, were considered as positive in the primary screening. Colposcopic findings were categorized as normal, low-grade lesions, high-grade lesions, or suspicious for cancer. Findings of low-grade lesions or worse were defined as abnormal colposcopy results. Histopathological diagnoses were classified as normal, low-grade squamous intraepithelial lesion (LSIL)/cervical intraepithelial neoplasia grade 1 (CIN1), HSIL/CIN2-3, adenocarcinoma *in situ* (AIS), micro-invasive cervical cancer, invasive cervical cancer, or inflammation/other non-neoplastic findings. The primary outcomes of interest were HSIL/CIN2–3 and more advanced cervical lesions. The outcome indicators are summarized in **Table 1**.

**Table 1:** Definition of outcome indicators.

Indicators	Formula
HPV infection rate	(Number of HPV positive cases / Number of participants tested for HPV) × 100%
Cytology triage rate	(Number of non-16/18 HR-HPV positive cases who actually underwent cytology triage / Total number of non-16/18 HR-HPV positive cases) × 100%
Abnormality rate of primary screening	[(HPV 16/18-positive cases + cases of non-16/18 HR-HPV positive combined with cytology triage results ≥ ASC-US) / Number of participants receiving HPV-based screening] × 100%
Attendance rate of colposcopy	(Number of cases with abnormal primary screening results who actually received colposcopy / Number of cases with abnormal primary screening results who needed to undergo colposcopy) ×100%
Colposcopy abnormality rate	(Number of cases with abnormal colposcopy findings / Number of cases who underwent colposcopy × 100%
Attendance rate of histopathology	(Number of cases who actually received histopathological diagnosis / Number of cases with abnormal colposcopy findings who needed to undergo histopathological diagnosis) ×100%
Detection rate of cervical precancerous lesions and cancer	(Number of cases with histopathologic findings ≥ HSIL/CIN2-3 / Number of total participants in screening) × 100,000
Early diagnosis rate	(Number of cases with histopathologic findings of HSIL/CIN2-3, AIS and micro-invasive cancer / Total number of cases with histopathologic findings ≥ HSIL/CIN2-3) × 100%

### 2.4 Statistical analysis

All statistical analyses were conducted using IBM SPSS Statistics version 29.0. For variables with missing values, multiple imputation was performed to ensure data completeness. Basic characteristics of participants, screening outcomes, and effectiveness indicators were summarized using frequencies and proportions. Chi-squared tests were used to compare differences in the prevalence of HPV infection and cervical cancer/precancerous lesions across participant characteristics. Multivariable logistic regression with the enter approach was performed to identify factors associated with HPV infection and cervical cancer/precancerous lesions, and the Wald test was used to assess statistical significance. All statistical tests conducted were two-sided with a statistically significance level of *P* < 0.05.

## 3 Results

### 3.1 Participant characteristics

Among the 107,120 women included in this study, the vast majority were Han Chinese (99.23%, 106,298/107,120), lived in suburban areas (89.21%, 95,567/107,120), and 54.90% (58,809/107,120) were under 50 years of age. Most women experienced menarche at ages 13-14 (51.31%, 54,959/107,120). A total of 40.66% (43,552/107,120) were postmenopausal, over half of whom (51.53%, 22,442/43,552) underwent menopause after the age of 50. Regarding reproductive history, 96.59% (103,467/107,120) had 0 to 2 deliveries, and 84.38% (90,386/107,120) had 0 to 2 abortions, with the first birth most commonly occurring between ages 21-25 (69.58%, 74,539/107,120). Only 25.72% (27,549/107,120) reported condom use in the past three years. Additionally, 2.05% (2,192/107,120) had a history of reproductive tract infections, and 0.83% (887/107,120) had a family history of cervical cancer (**Table 2**).

**Table 2:** Characteristics of screening population (*n* =107,120).

Characteristics		*n*	%
Age (years)	35-39	18,476	17.25
	40-44	17,574	16.41
	45-49	22,759	21.25
	50-54	26,136	24.40
	55-59	17,491	16.33
	60-64	4,684	4.37
Ethnicity	Han	106,298	99.23
	Others	822	0.77
Residential area	Urban core areas	11,553	10.79
	Suburban areas	95,567	89.21
Age of menarche (years)	≤12	28,312	26.43
	13-14	54,959	51.31
	≥15	23,849	22.26
Menopausal status	Yes	43,552	40.66
	No	61,349	57.27
	Uncertainty	2,219	2.07
Age of menopause (years)	≤39	545	1.25
	40-44	3,089	7.09
	45-49	17,476	40.13
	≥50	22,442	51.53
Number of deliveries	0-2	103,467	96.59
	≥3	3,653	3.41
Number of abortions	0-2	90,386	84.38
	≥3	16,734	15.62
Age of first birth (years)	≤20	12,544	11.71
	21-25	74,539	69.58
	≥26	20,037	18.71
Condom use	Yes	27,549	25.72
	No	79,571	74.28
Past history of reproductive tract infections	Yes	2,192	2.05
	No	104,928	97.95
Family history of cervical cancer	Yes	887	0.83
	No	106,233	99.17

Urban core areas refer to the six central districts of Chengdu: Wuhou, Jinjiang, High-tech Zone, Chenghua, Qingyang, and Jinniu.

### 3.2 Cervical cancer screening outcomes

#### 3.2.1 Gynecological examination findings

All 107,120 women underwent routine gynecological examinations, and valid results were obtained. Among them, 72.07% (77,204/107,120) had normal examination findings. The prevalences of bacterial vaginitis (BV), vulvovaginal candidiasis (VVC), trichomonas vaginitis (TV), and non-specific vaginitis were 1.48% (1,589/107,120), 1.47% (1,572/107,120), 0.20% (213/107,120), and 0.01% (13/107,120), respectively. Additionally, other gynecological conditions, including chronic cervicitis, mucopurulent cervicitis, cervical polyps, uterine fibroids, and pelvic and adnexal diseases, were observed in 25.42% (27,227/107,120) of the population (**Table 3**).

**Table 3:** Gynecological examinations results (*n* =107,120).

Gynecological examinations results	*n*	%
Normal	77,204	72.07
Bacterial vaginitis	1,589	1.48
Vulvovaginal candidiasis	1,572	1.47
Trichomonas vaginitis	213	0.20
Non-specific vaginitis	13	0.01
Other gynecological conditions	27,227	25.42

#### 3.2.2 HR-HPV testing and cytology triage outcomes

**Figure 2:**
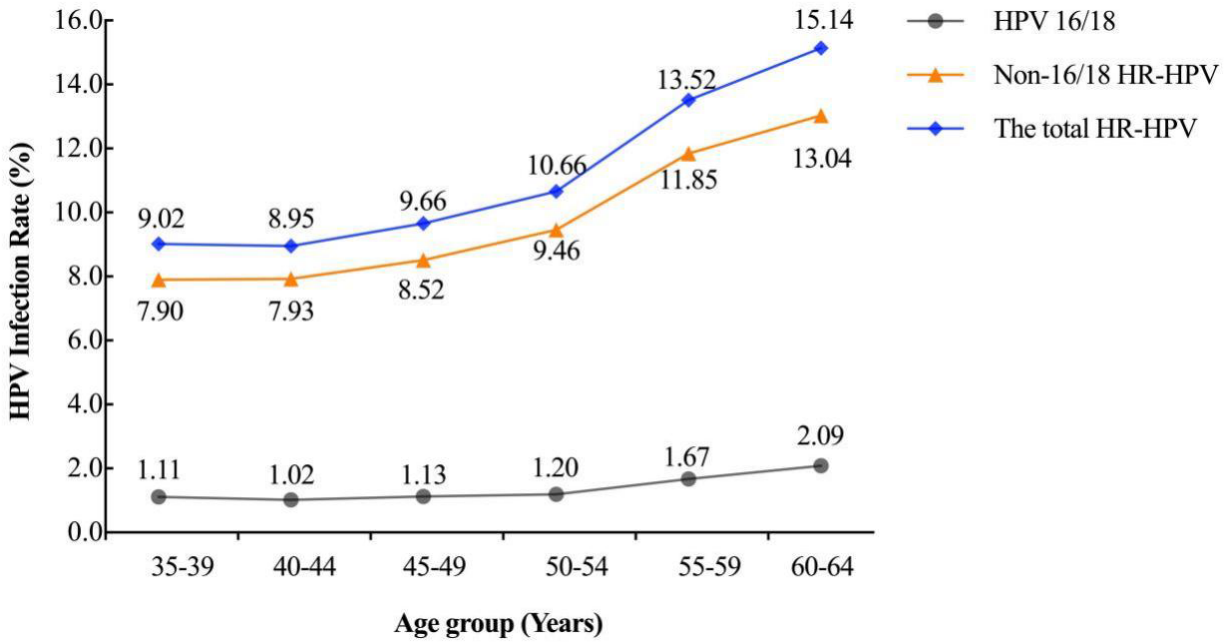
Age-specific prevalence of the HR-HPV infection.

The overall rate of HR-HPV infection among participants undergoing HPV testing was 10.54% (11,295/107,120). Specifically, the rate of HPV 16/18 infection was 1.26% (1,346/107,120), while the rate of non-16/18 HR-HPV infection was 9.29% (9,949/107,120). The HR-HPV infection rates showed overall increasing trends with age (*P*<0.001) (**Figure 2**).

Among the 9,949 participants positive for non-16/18 HR-HPV, 87.86% (8,741/9,949) underwent cytology triage, while 12.14% (1,208/9,949) had no records in the database. The positive rate for cytology triage findings classified as ≥ASC-US was 23.02% (2,012/8,741) (**Table 4**). Overall, 3,358 cases involved either HPV 16/18 infection or non-16/18 HR-HPV with cytology triage findings classified as ≥ASC-US, resulting in a primary screening abnormality rate of 3.13% (3,358/107,120).

**Table 4:** Cytology triage findings (*n* =8,741).

TBS classification diagnostic criteria	*n*	%
NILM	6,729	76.98
ASC-US	1,301	14.88
ASC-H	91	1.04
LSIL	438	5.01
HSIL	176	2.01
SCC	0	0.00
AGC	6	0.07
AIS	0	0.00
ADC	0	0.00

TBS, the Bethesda system; NlLM, negative for intraepithelial lesion or malignancy; ASC-US, atypical squamous cells of undetermined significance; ASC-H, atypical squamous cells, cannot exclude high-grade squamous intraepithelial lesion; LSIL, low-grade squamous intraepithelial lesion; HSIL, high-grade squamous intraepithelial lesion; SCC, squamous cell carcinoma; AGC, atypical glandular cells; AIS, adenocarcinoma *in situ*; ADC, adenocarcinoma.

#### 3.2.3 Colposcopy referral and histopathological diagnosis

A total of 3,358 women with abnormal primary screening results should be referred for colposcopy, but only 81.95% (2,752/3,358) underwent the procedure. The main reasons for non-completion included loss to follow-up (77.06%, 467/606), refusal (18.98%, 115/606), and undergoing colposcopy elsewhere without reporting the results (3.96%, 24/606). Among those examined, 76.24% (2,098/2,752) had low-grade lesions or worse (**Table 5**). Of these, 94.61% (1,985/2,098) underwent histopathological examination, while others were lost to follow-up (87.61%, 99/113), refused biopsy (7.08%, 8/113), or had invalid tissue samples (5.31%, 6/113). In total, 428 cases of HSIL/CIN2–3 or more severe lesions were detected (**Table 5**).

**Table 5:** Colposcopy referral and histopathological diagnosis results.

Results	*n*	%
Colposcopic findings (*n* = 2,752)		
Normal	654	23.76
LSIL	1,514	55.01
HSIL	295	10.72
Suspected cancer	42	1.53
Other	247	8.98
Histopathological findings (*n* = 1,985)		
Normal	17	0.86
LSIL/CIN1	925	46.60
HSIL/CIN2-3	405	20.40
AIS	2	0.10
Micro-invasive cervical cancer	11	0.55
Invasive cervical cancer	10	0.50
Inflammation or other	615	30.98

LSIL, low-grade squamous intraepithelial lesion; HSIL, high-grade squamous intraepithelial lesion; CIN, cervical intraepithelial neoplasia; AIS, adenocarcinoma *in situ*.

#### 3.2.4 Detection of cervical cancer and precancerous lesions

A total of 428 patients with high-grade cervical precancerous lesions or cervical cancer were identified among the 107,120 individuals. This included 405 cases of HSIL/CIN2-3, 2 cases of AIS, 11 cases of micro-invasive cervical cancer, and 10 cases of invasive cervical cancer (**Table 5**). The overall detection rate of cervical cancer and precancerous lesions was 399.55 per 100,000. Specifically, the detection rates was 19.60 per 100,000 for cervical cancer (micro-invasive and invasive cases only) and 379.95 per 100,000 for high-grade precancerous lesions (HSIL/CIN2–3 and AIS). The early diagnosis rate of cervical cancer screening was 97.66% (418/428).

**Figure 3:**
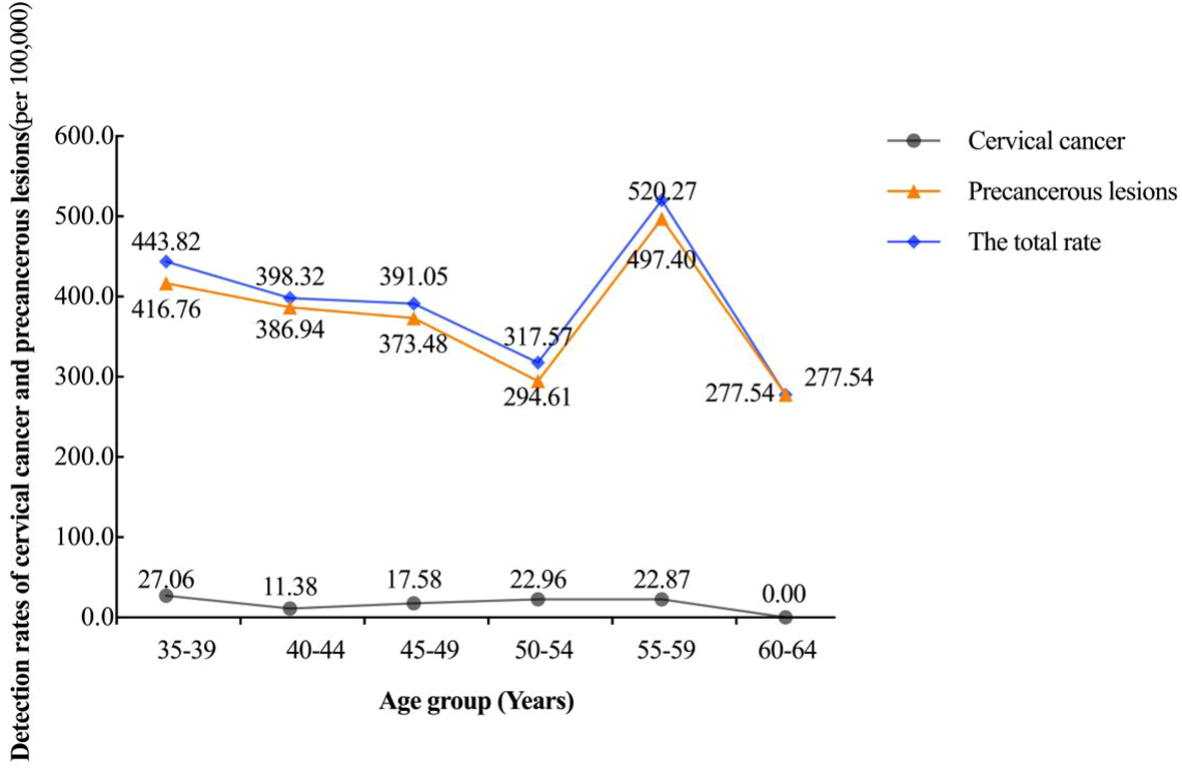
Age-specific trends in the detection rates of cervical cancer and precancerous lesions.

This study analyzed the age-specific trends in the detection rates of cervical cancer and precancerous lesions (**Figure 3**). The detection rates of precancerous lesions and the overall detection rate (including both cervical cancer and precancerous lesions) generally declined with age (*P* = 0.020 and 0.019, respectively). However, a secondary peak was observed in the 55–59 age group, with an overall detection rate of 520.27 per 100,000 and a precancerous lesion detection rate of 497.40 per 100,000. In contrast, the detection rate of cervical cancer remained relatively low across all age groups, with slight fluctuations, peaking at 27.06 per 100,000 in the 35–39 age group.

**Figure 4:**
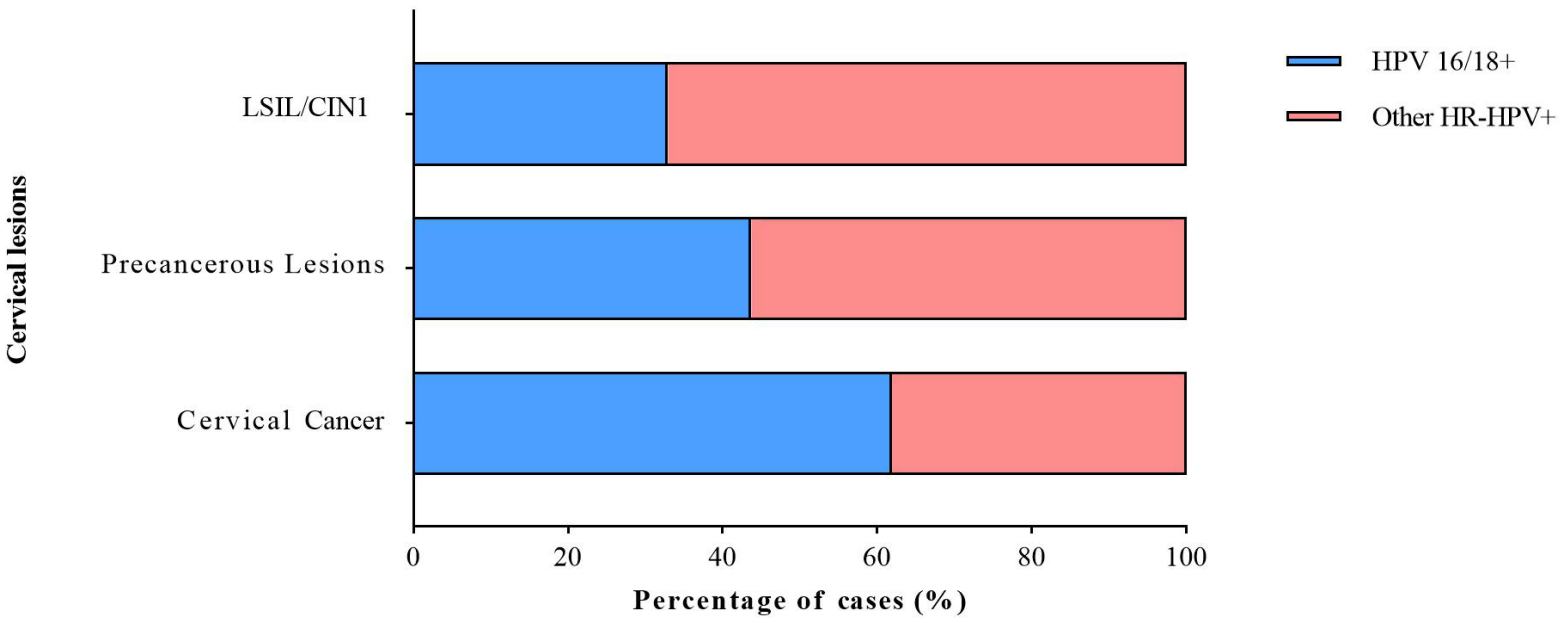
Distribution of HR-HPV infections in cases with cervical lesions.

Additionally, we analyzed the distribution of HR-HPV infections among individuals with LSIL/CIN1, precancerous lesions, and cervical cancer. The proportion of HPV 16/18 infections increased with disease severity, being highest in cervical cancer cases (61.90%, 13/21), followed by precancerous lesions (43.73%, 178/407) and LSIL/CIN1 (32.97%, 305/925). In contrast, other HR-HPV infections were more prevalent in lower-grade lesions (**Figure 4**).

### 3.3 Risk factors associated with HR-HPV infection and cervical cancer/precancerous lesions

#### 3.3.1 Univariate analysis

Among the 107,120 women who participated in the cervical cancer screening program, 11,295 tested positive for HR-HPV. A total of 105,193 women completed the full screening process with valid results, and 428 cases of high-grade cervical precancerous lesions or cervical cancer were identified. Univariate analysis showed that older age, residence in suburban areas, later menarche, postmenopausal status, higher numbers of deliveries and abortions, younger age at first birth, and lack of condom use were all associated with a higher likelihood of HR-HPV infection (all *P* < 0.05). Moreover, the prevalence of cervical cancer/precancerous lesions differed significantly across age groups, residential areas, and menopausal statuses (all *P* < 0.05) (**Table 6**).

**Table 6:** Univariate analysis of risk factors associated with HR-HPV infection and cervical cancer/precancerous lesions.

Characteristics	HR-HPV infection			Cervical cancer/precancerous lesions		
	Yes [*n* (%)]	No [*n* (%)]	*P*	Yes [*n* (%)]	No [*n* (%)]	*P*
Age (years)			**<0.001**			**0.018**
35-39	1,666 (9.02)	16,810 (90.98)		82 (0.45)	18,149 (99.55)	
40-44	1,573 (8.95)	16,001 (91.05)		70 (0.40)	17,240 (99.60)	
45-49	2,198 (9.66)	20,561 (90.34)		89 (0.40)	22,300 (99.60)	
50-54	2,785 (10.66)	23,351 (89.34)		83 (0.32)	25,586 (99.68)	
55-59	2,364 (13.52)	15,127 (86.48)		91 (0.53)	16,966 (99.47)	
60-64	709 (15.14)	3,975 (84.86)		13 (0.29)	4,524 (99.71)	
Residential area			**<0.001**			**0.003**
Urban core areas	1,023 (8.85)	10,530 (91.15)		27 (0.24)	11,276 (99.76)	
Suburban areas	10,272 (10.75)	85,295 (89.25)		401 (0.43)	93,489 (99.57)	
Ethnicity			0.791			0.674
Han	11,206 (10.54)	95,092 (89.46)		426 (0.41)	103,967 (99.59)	
Others	89 (10.83)	733 (89.17)		2 (0.25)	798 (99.75)	
Age of menarche (years)			**0.017**			0.962
≤12	2,927 (10.34)	25,385 (89.66)		116 (0.42)	27,791 (99.58)	
13-14	5,735 (10.44)	49,224 (89.56)		217 (0.40)	53,678 (99.60)	
≥15	2,633 (11.04)	21,216 (88.96)		95 (0.41)	23,296 (99.59)	
Menopausal status			**<0.001**			**0.047**
Yes	5,381 (12.36)	38,171 (87.64)		168 (0.39)	42,443 (99.61)	
No	5,696 (9.28)	55,653 (90.72)		258 (0.43)	60,148 (99.57)	
Uncertainty	218 (9.82)	2,001 (90.18)		2 (0.09)	2,174 (99.91)	
Age of menopause (years)			0.077			0.087
≤39	85 (15.60)	460 (84.40)		2 (0.38)	527 (99.62)	
40-44	402 (13.01)	2,687 (86.99)		20 (0.66)	3,003 (99.34)	
45-49	2,144 (12.27)	15,332 (87.73)		59 (0.34)	17,053 (99.66)	
≥50	2,750 (12.25)	19,692 (87.75)		87 (0.40)	21,862 (99.60)	
Number of deliveries			**<0.001**			0.127
0-2	10,825 (10.46)	92,642 (89.54)		408 (0.40)	101,263 (99.60)	
≥3	470 (12.87)	3,183 (87.13)		20 (0.57)	3,502 (99.43)	
Number of abortions			**0.001**			0.624
0-2	9,407 (10.41)	80,979 (89.59)		358 (0.40)	88,531 (99.60)	
≥3	1,888 (11.28)	14,846 (88.72)		70 (0.43)	16,234 (99.57)	
Age of first birth (years)			**<0.001**			0.385
≤20	1,500 (11.96)	11,044 (88.04)		58 (0.47)	12,224 (99.53)	
21-25	7,999 (10.73)	66,540 (89.27)		286 (0.39)	72,860 (99.61)	
≥26	1,796 (8.96)	18,241 (91.04)		84 (0.42)	19,681 (99.58)	
Condom use			**<0.001**			0.280
Yes	2,362 (8.57)	25,187 (91.43)		101 (0.37)	27,126 (99.63)	
No	8,933 (11.23)	70,638 (88.77)		327 (0.42)	77,639 (99.58)	
Past history of reproductive tract infections			0.069			0.424
Yes	257 (11.72)	1,935 (88.28)		11 (0.52)	2,121 (99.48)	
No	11,038 (10.52)	93,890 (89.48)		417 (0.40)	102,644 (99.60)	
Family history of cervical cancer			0.781			0.587
Yes	91 (10.26)	796 (89.74)		2 (0.23)	861 (99.77)	
No	11,204 (10.55)	95,029 (89.45)		426 (0.41)	103,904 (99.59)	

Urban core areas refer to the six central districts of Chengdu: Wuhou, Jinjiang, High-tech Zone, Chenghua, Qingyang, and Jinniu.

Bold values indicate statistical significance (P < 0.05).

#### 3.3.2 Multivariable logistic regression analysis

As shown in **Table 7**, multivariable logistic regression analysis indicated that age ≥55 years (55–59 years: aOR=1.34, 95% CI: 1.22-1.47; 60–64 years: aOR=1.53, 95% CI: 1.37-1.73), residence in suburban areas (aOR=1.19, 95% CI: 1.11-1.27), menopause (aOR=1.08, 95% CI: 1.01-1.15), having three or more childbirths (aOR=1.18, 95% CI: 1.06-1.31), and three or more abortions (aOR=1.16, 95% CI: 1.06-1.26) were associated with a higher risk of HR-HPV infection. In contrast, later age at first birth (21–25 years: aOR=0.88, 95% CI: 0.83-0.94; ≥26 years: aOR=0.80, 95% CI: 0.74-0.86) and condom use (aOR=0.87, 95% CI: 0.82-0.92) were protective factors. In addition, age (55–59 years: aOR=1.57, 95% CI: 1.03-2.41), residential areas (suburban areas: aOR=1.82, 95% CI: 1.23-2.69), and menopausal status (yes or uncertainty: aOR=0.72, 95% CI: 0.52-0.99) were also identified as factors associated with cervical cancer/precancerous lesions.

**Table 7:** Multivariable analysis of risk factors associated witd HR-HPV infection and cervical cancer/precancerous lesions.

Characteristics	*β*	*SE*	*P*	aOR (95%CI)
HR-HPV infection				
Age (years)				
35-39 (ref)				1.00
40-44	-0.035	0.037	0.350	0.97 (0.90, 1.04)
45-49	0.009	0.036	0.809	1.01 (0.94, 1.08)
50-54	0.057	0.041	0.160	1.06 (0.98, 1.15)
55-59	0.295	0.047	**<0.001**	1.34 (1.22, 1.47)
60-64	0.436	0.059	**<0.001**	1.55 (1.38, 1.74)
Residential area				
Urban core areas (ref)				1.00
Suburban areas	0.169	0.035	**<0.001**	1.19 (1.11, 1.27)
Age of menarche (years)				
≤12 (ref)				1.00
13-14	-0.019	0.024	0.438	0.98 (0.94, 1.03)
≥15	-0.038	0.029	0.188	0.96 (0.91, 1.02)
Menopausal status				
No (ref)				1.00
Yes	0.076	0.033	**0.022**	1.08 (1.01, 1.15)
Uncertainty	-0.059	0.075	0.429	0.94 (0.82, 1.09)
Number of deliveries				
0-2 (ref)				1.00
≥3	0.148	0.051	**0.004**	1.16 (1.05, 1.28)
Number of abortions				
0-2 (ref)				1.00
≥3	0.118	0.027	**<0.001**	1.13 (1.07, 1.19)
Age of first birtd (years)				
≤20 (ref)				1.00
21-25	-0.121	0.030	**<0.001**	0.89 (0.84, 0.94)
≥26	-0.226	0.038	**<0.001**	0.80 (0.74, 0.86)
Condom use				
No (ref)				1.00
Yes	-0.140	0.027	**<0.001**	0.87 (0.82, 0.92)
Cervical cancer/precancerous lesions				
Age (years)				
35-39 (ref)				1.00
40-44	-0.109	0.163	0.506	0.90 (0.65, 1.24)
45-49	-0.090	0.156	0.563	0.91 (0.67, 1.24)
50-54	-0.141	0.187	0.453	0.87 (0.60, 1.25)
55-59	0.454	0.218	**0.037**	1.57 (1.03, 2.41)
60-64	-0.131	0.339	0.699	0.88 (0.45, 1.70)
Residential area				
Urban core areas (ref)				1.00
Suburban areas	0.598	0.200	**0.003**	1.82 (1.23, 2.69)
Menopausal status				
No (ref)				1.00
Yes or uncertainty	-0.335	0.213	**0.040**	0.72 (0.52, 0.99)

Urban core areas refer to tde six central districts of Chengdu: Wuhou, Jinjiang, High-tech Zone, Chenghua, Qingyang, and Jinniu. SE, standard error; aOR, adjusted odds ratio; CI, confidence interval.

Bold values indicate statistical significance (P < 0.05).

#### 3.3.3 Stratified analysis by menopausal status

To further examine whether menopausal status modified the observed associations, stratified multivariable logistic regression analyses were performed according to menopausal status. Women with uncertain menopausal status were combined with the postmenopausal group, as their physiological characteristics were considered more comparable to those of postmenopausal women. Moreover, when cervical cancer/precancerous lesions were analyzed as the outcome, the number of cases within each stratum was relatively small. To ensure sufficient sample size and enhance model stability, the original six age categories were collapsed into three groups: 35-44, 45-54, and 55–64 years.

Stratified analyses by menopausal status are presented in **Table 8**. Among premenopausal women, older age (55–59 years: aOR =1.30, 95% CI: 1.00-1.69), residence in suburban areas (aOR =1.16, 95% CI: 1.06-1.26), and having three or more abortions (aOR =1.12, 95% CI: 1.04-1.20) were associated with an increased risk of HR-HPV infection, whereas later age at first birth (21–25 years: aOR =0.88, 95% CI: 0.81-0.96; ≥26 years: aOR =0.81, 95% CI: 0.73-0.89) and condom use (aOR =0.87, 95% CI: 0.82-0.92) were protective. Residence in suburban areas (aOR =1.85, 95% CI: 1.16-2.95) was also a risk factor for cervical cancer/precancerous lesions.

**Table 8:** Multivariable analysis of risk factors associated witd HR-HPV infection and cervical cancer/precancerous lesions by menopausal status.

Characteristics	Premenopausal		Postmenopausal	
	*P*	aOR(95%CI)	*P*	aOR(95%CI)
HR-HPV infection				
Age (years)				
35-39 (ref)		1.00		1.00
40-44	0.384	0.97 (0.90, 1.04)	0.677	0.88 (0.48, 1.61)
45-49	0.411	1.03 (0.96, 1.11)	0.756	0.92 (0.54, 1.56)
50-54	0.774	1.01 (0.92, 1.11)	0.809	1.07 (0.63, 1.80)
55-59	**0.049**	1.30 (1.00, 1.69)	0.271	1.34 (0.80, 2.26)
60-64	0.542	1.30 (0.56, 3.06)	0.102	1.55 (0.92, 2.62)
Residential area				
Urban core areas (ref)		1.00		1.00
Suburban areas	**0.002**	1.16 (1.06, 1.26)	**<0.001**	1.23 (1.11, 1.38)
Age of menarche (years)				
≤12 (ref)		1.00		1.00
13-14	0.756	0.99 (0.93, 1.06)	0.382	0.97 (0.90, 1.04)
≥15	0.753	0.99 (0.91, 1.07)	0.111	0.94 (0.87, 1.02)
Number of deliveries				
0-2 (ref)		1.00		1.00
≥3	0.061	1.16 (0.99, 1.35)	**0.027**	1.17 (1.02, 1.33)
Number of abortions				
0-2 (ref)		1.00		1.00
≥3	**0.002**	1.12 (1.04, 1.20)	**0.002**	1.14 (1.05, 1.23)
Age of first birtd (years)				
≤20 (ref)		1.00		1.00
21-25	**0.003**	0.88 (0.81, 0.96)	**0.007**	0.89 (0.82, 0.97)
≥26	**<0.001**	0.81 (0.73, 0.89)	**<0.001**	0.78 (0.70, 0.88)
Condom use				
No (ref)		1.00		1.00
Yes	**<0.001**	0.87 (0.82, 0.92)	**0.033**	0.87 (0.76, 0.99)
Cervical cancer/precancerous lesions				
Age (years)				
35-44 (ref)		1.00		1.00
45-54	0.745	0.96(0.75, 1.23)	0.873	0.89 (0.22, 3.65)
55-64	0.826	1.14 (0.36, 3.58)	0.573	1.50 (0.37, 6.08)
Residential area				
Urban core areas (ref)		1.00		1.00
Suburban areas	**0.010**	1.85 (1.16, 2.95)	0.100	1.82 (0.89, 3.70)

Urban core areas refer to tde six central districts of Chengdu: Wuhou, Jinjiang, High-tech Zone, Chenghua, Qingyang, and Jinniu. SE, standard error; aOR, adjusted odds ratio; CI, confidence interval.

Bold values indicate statistical significance (P < 0.05).

Among postmenopausal women, HR-HPV infection was more likely in those residing in suburban areas (aOR =1.23, 95% CI: 1.11-1.38), with three or more childbirths (aOR =1.17, 95% CI: 1.02-1.33), or three or more abortions (aOR =1.14, 95% CI: 1.05-1.23), whereas later age at first birth (21–25 years: aOR =0.89, 95% CI: 0.82-0.97; ≥26 years: aOR =0.78, 95% CI: 0.70-0.88) and condom use (aOR =0.87, 95% CI: 0.76-0.99) were protective. No factors were significantly associated with cervical cancer/precancerous lesions in this group.

## 4 Discussion

Persistent infection with HR-HPV is widely recognized as the main cause of cervical cancer. Globally, HPV is detected in 99.7% of cervical cancer cases (3, 4). In China, the prevalence of HPV among women varies substantially across regions, ranging from approximately 6.2% to 31.3% (5). In our screened population, the HR-HPV prevalence was 10.54%, aligning with previous report from Sichuan Province (12.6%) and the national average (11.9%) (5, 11). HPV types 16 and 18 are considered the primary oncogenic types, accounting for the majority of HR-HPV infections in Europe and America (12, 13). In contrast, the prevalence of HPV 16/18 in our population was only 1.26%, representing 11.92% of all HR-HPV-positive cases, reflecting regional differences in HPV epidemiology. Notably, in Sichuan and other parts of China, such as Shanghai and Hong Kong, HPV types 52 and 58 have been reported as predominant high-risk types (14–16). It should be noted that the HPV genotyping kits employed in Chengdu’s cervical cancer screening program only provide separate detection reports for types 16 and 18, limiting our ability to further characterize the distribution of other high-risk types in the screening population.

In this study, the detection rate of cervical cancer was 19.60 per 100,000, and that of high-grade precancerous lesion was 379.95 per 100,000. The cervical cancer incidence observed in this study was slightly higher than the global average (13.3 per 100,000) and rates reported in high-income countries (9.1-12.7 per 100,000) (17), but lower than that reported in some low-income countries (27.2 per 100,000) (17, 18). Within China, the observed cervical cancer prevalence in our study was also higher than the national average (10.7 per 100,000) and that reported in some eastern regions (17, 19), while the detection rate of high-grade precancerous lesions was also higher than in Beijing (approximately 70 per 100,000) and Xinjiang (approximately 110 per 100,000) (12, 20). This disparity may be associated with differences in local HPV infection patterns, screening coverage, and the sensitivity of detection methods. Previous studies have shown that combining HPV testing with cervical cytology significantly enhances the detection of cervical cancer and precancerous lesions (21), and our findings further support this conclusion. The high detection rate of precancerous lesions in this study highlights not only the effectiveness of the current screening strategy but also underscores its essential role in the early identification and timely management of cervical abnormalities.

Additionally, this study identified several risk factors associated with HR-HPV infection and cervical cancer/precancerous lesions, including age, residential area, menopausal status, reproductive history, and condom use.

Firstly, our findings indicated that women aged ≥55 years had an increased risk of HR-HPV infection, with the risk progressively increasing with age. Specifically, compared to women aged 35–39 years, those aged 55–59 and 60–64 years had a 34.0% and 53.4% higher risk of infection, respectively. This increased susceptibility may be associated with age-related declines in physical condition and immune function, resulting in reduced viral clearance efficiency, prolonged infection duration, and higher likelihood of persistent infection (22, 23). Moreover, postmenopausal women exhibited a higher risk of HR-HPV infection compared to premenopausal women, likely due to disruptions in reproductive endocrine and immune regulation during the peri- and postmenopausal periods. Previous studies have shown that menopause leads to reduced hypothalamic-pituitary-gonadal (HPG) axis function, decreased ovarian activity, and lower secretion of sex hormones, particularly estrogen (24). These hormonal changes induce physiological alterations in the reproductive tract, including thinning and atrophy of the vaginal mucosa, decreased glycogen content in the epithelial cells, and increased vaginal pH, which disrupt the vaginal microenvironment and weaken local immune defenses, thereby facilitating HPV entry and persistence (25, 26). Clinical evidence also supports the role of estrogen therapy in enhancing HPV clearance and shortening the time to viral clearance in postmenopausal women (27). Furthermore, cervical structural changes during menopause, such as cervical atrophy, squamocolumnar junction migration into the endocervical canal, epithelium thinning, and increased potential for cellular atypia, may create favorable conditions for HPV infection and persistence (28, 29). Limited awareness of HPV and related diseases, together with lower health consciousness and preventive behaviors among older women, may further contribute to the elevated risk (30). Stratified analysis further revealed that among premenopausal women, the risk of HR-HPV infection was significantly increased in the 55–59 age group, whereas the effect of age was no longer significant among postmenopausal women. This suggests a potential interaction between age and menopausal status, where menopause-related physiological changes may become the predominant factors influencing infection risk in older women, attenuating the effect of chronological age. Additionally, in this study, the overall detection rate of cervical cancer and precancerous lesions decreased with age, with the lowest detection rates observed in the 50–54 and 60–64 age groups. Notably, a secondary peak occurred in the 55–59 age group, suggesting age-specific risk factors. Meanwhile, peri- and postmenopausal women showed a lower risk compared to premenopausal women. This pattern differs from that of HR-HPV infection, suggesting that the development of cervical lesions may not only be influenced by infection status, but also by multiple factors such as host-related factors and screening behaviors, which warrants further investigation.

Secondly, residing in suburban areas emerged as an independent risk factor for HR-HPV infection and cervical cancer or precancerous lesions, consistent with findings from northern China (31, 32). The majority of participants in our study (89.21%) resided in suburban or resource-limited areas. Compared to women in central urban districts, those in these areas often have limited access to healthcare resources, resulting in lower rates of regular screening and delayed medical intervention, which may lead to the persistence of HR-HPV infection and the progression of cervical lesions (33–35). Moreover, low levels of health awareness in this population, especially in the knowledge of HPV, vaccination and cervical cancer prevention, may further increase their risk of HR-HPV infection and cervical cancer (34, 36). Socioeconomic disadvantages may also restrict access to high-quality healthcare services (35).

Thirdly, multiparity (≥3 births) and having three or more abortions were identified as risk factors for HR-HPV infection in this study, whereas a later age at first birth was associated with a lower risk of infection. Previous studies suggest that hormonal and immune fluctuations during pregnancy can selectively reactivate latent HPV infections, including HPV 16 and other HR-HPV types, thereby increasing susceptibility and persistence of infection in pregnant women (37, 38). Both childbirth and induced abortion can cause mechanical damage to cervical and vaginal tissues, compromising the local defense mechanisms and increasing cervical vulnerability to infections (39, 40). A higher number of deliveries and abortions may therefore lead to more frequent cervical trauma and repeated hormonal fluctuations, cumulatively weakening the cervical barrier and elevating the risk of HPV infection. Stratified analysis indicated that, among premenopausal women, a history of multiple abortions was associated with HR-HPV infection, whereas in postmenopausal women, both multiparity and multiple abortions were risk factors. This pattern suggests that, with advancing age and changes in physiological status, reproductive history may exert a cumulative effect on cervical susceptibility to HR-HPV infection. However, this study could not distinguish between induced and spontaneous abortions, and the potential differential impact of abortion methods on HPV infection warrants further investigation. A later age at first birth may indicate a later onset of sexual activity and HPV exposure, by which time the cervix is more mature and resistant to external pathogens. Moreover, women with a later first birth often have fewer pregnancies overall, further reducing their risk of HPV infection (41).

Lastly, condom use was identified as a protective factor against HR-HPV infection, with a 13.1% reduction in risk. Previous studies reported stronger protective effects, with Sui et al. and Bai et al. observing reduction of 64% and 67%, respectively (42, 43). Since HR-HPV is mainly transmitted through sexual contact, consistent condom use helps prevent direct cervical exposure to HPV while also reducing stimulation from other infectious agents, physical trauma, and semen, which lowers the occurrence of cervical cell dysplasia and inflammation, and ultimately reducing the risk of HR-HPV infection (44, 45).

Based on the findings of this study, we recommend strengthening cervical cancer prevention efforts, particularly among women in suburban or resource-limited areas, who are at higher risk for HR-HPV infection and cervical lesions. Screening services should be reinforced through the integration of primary healthcare facilities and community health centers to improve accessibility and ensure continuity of care. Women of advanced age, especially those in the perimenopausal or postmenopausal period, as well as those with a history of multiparity or multiple abortions should be prioritized for individualized follow-up. Additionally, health education efforts should be intensified through community and media campaigns to improve awareness of HPV, vaccination, and cervical cancer prevention, while promoting protective behaviors such as condom use and appropriate reproductive planning. These targeted strategies may contribute to more effective prevention, earlier detection, and timely intervention for cervical cancer.

This study has several limitations. Firstly, examinations and tests were performed across multiple hospitals, primary maternal and child health institutions, and third-party laboratories, which may have introduced measurement and reporting biases despite rigorous quality control. Additionally, some participants were lost to follow-up, refused examinations, or provided invalid samples, all of which could contribute to selection and information bias. Secondly, the screening registration database did not fully record information on several important sociodemographic and behavioral characteristics, such as marital status, education, income, occupation, sexual behavior, lifestyle habits, and HPV vaccination history. The lack of these variables restricted the scope of our analysis and may have limited the comprehensiveness of assessing potential influencing factors (23). Furthermore, the HPV genotyping kits used in the current screening program in Chengdu only reported HPV-16 and HPV-18 separately, while other high-risk types were grouped together. This limited the ability to assess genotype-specific risks and their implications for surveillance and prevention. Broader genotyping in future screening could improve understanding of local HPV type distribution and support more targeted vaccine strategies. Finally, the reasons for loss to follow-up among individuals who did not complete cytology triage, colposcopy, or histopathological examination could not be further analyzed. Despite these constraints, our results may still provide valuable information and insights.

## 5 Conclusion

The overall prevalence of HR-HPV infection among women aged 35–64 in southwestern China was 10.54%. The crude incidence rate of cervical cancer was 19.60 per 100,000, highlighting a significant disease burden in this population. Age ≥55 years, suburban residence, menopausal status, multiparity, and a history of multiple abortions were identified as significant risk factors for HR-HPV infection. Protective factors included condom use and later age at first birth. Additionally, age, residential area, and menopausal status were also significantly associated with the occurrence of cervical cancer and precancerous lesions. This study has identified key target populations for screening and elucidated influencing factors for HR-HPV infection, cervical cancer, and cervical precancerous lesions, providing evidence to optimize screening strategies and implement precision prevention and control measures.

## Data Availability

The datasets presented in this article are not readily available because Due to the sensitivity of the data, for further inquiries about the original dataset, please contact the Maternal and Child Health Department of the Chengdu Municipal Health Commission (cdfyjkfwc@163.com) for consent and authorization before providing the raw data. Requests to access the datasets should be directed to the Maternal and Child Health Department of the Chengdu Municipal Health Commission (cdfyjkfwc@163.com).
